# Measuring the Impact of an Open Web-Based Prescribing Data Analysis Service on Clinical Practice: Cohort Study on NHS England Data

**DOI:** 10.2196/10929

**Published:** 2019-01-16

**Authors:** Alex J Walker, Helen J Curtis, Richard Croker, Seb Bacon, Ben Goldacre

**Affiliations:** 1 Evidence Based Medicine DataLab Nuffield Department of Primary Care Health Sciences University of Oxford Oxford United Kingdom

**Keywords:** drug prescribing, cost control, patient safety, treatment efficacy

## Abstract

**Background:**

OpenPrescribing is a freely accessible service that enables any user to view and analyze the National Health Service (NHS) primary care prescribing data at the level of individual practices. This tool is intended to improve the quality, safety, and cost-effectiveness of prescribing.

**Objective:**

We aimed to measure the impact of OpenPrescribing being viewed on subsequent prescribing.

**Methods:**

Having preregistered our protocol and code, we measured three different metrics of prescribing quality (mean percentile across 34 existing OpenPrescribing quality measures, available “price-per-unit” savings, and total “low-priority prescribing” spend) to see whether they changed after the viewing of Clinical Commissioning Group (CCG) and practice pages. We also measured whether practices whose data were viewed on OpenPrescribing differed in prescribing, prior to viewing, compared with those who were not. We used fixed-effects and between-effects linear panel regression to isolate change over time and differences between practices, respectively. We adjusted for the month of prescribing in the fixed-effects model to remove underlying trends in outcome measures.

**Results:**

We found a reduction in available price-per-unit savings for both practices and CCGs after their pages were viewed. The saving was greater at practice level (−£40.42 per thousand patients per month; 95% CI −54.04 to −26.81) than at CCG level (−£14.70 per thousand patients per month; 95% CI −25.56 to −3.84). We estimate a total saving since launch of £243 thosand at practice level and £1.47 million at CCG level between the feature launch and end of follow-up (August to November 2017) among practices viewed. If the observed savings from practices viewed were extrapolated to all practices, this would generate £26.8 million in annual savings for the NHS, approximately 20% of the total possible savings from this method. The other two measures were not different after CCGs or practices were viewed. Practices that were viewed had worse prescribing quality scores overall prior to viewing.

**Conclusions:**

We found a positive impact from the use of OpenPrescribing, specifically for the class of savings opportunities that can only be identified by using this tool. Furthermore, we show that it is possible to conduct a robust analysis of the impact of such a Web-based service on clinical practice.

## Introduction

The OpenPrescribing.net project aims to make prescribing data more accessible and impactful for clinicians, policy makers, and others. It does this through providing a user-friendly Web interface. It is hoped that this will enable safer, more effective, and cost-efficient prescribing by making users more aware of their prescribing behavior through comparisons with peers and by highlighting meaningful changes over time. OpenPrescribing gives a range of specific analyses and tools for each Clinical Commissioning Group (CCG) and individual primary care practice in three broad classes (set out in Textbox 1). Use of OpenPrescribing is driven largely by it being openly accessible and free to use; the service had 70,000 unique users over the past year. Broadly, users are obtained through word of mouth and social media engagement combined with some press coverage. Use of the service is not mandatory or associated with any payment in any locations that we are aware of.

There are many commercial tools that aim to improve prescribing in the United Kingdom. These include Oracle [3], Optum (ScriptSwitch) [4], Prescribing Services [5], and Prescribing Support Services [6]. While these tools may be effective at generating change, there is little publicly available evidence of robust testing. Such testing is an important element of good commissioning practice, in order to ensure that resources are used cost-effectively [7].

We set out to deliver a robust quantitative evaluation of the impact of the use of an open Web-based tool that may act as a template for other evaluations of similar tools, both in terms of the open and reproducible approach and the methodology used. We set out to measure whether any change in prescribing behavior occurs after the use of OpenPrescribing using the most robust observational methods to determine whether there is any causal association. Because it is possible that practices and CCGs who engage with their prescribing data are systematically different to those who do not, we also set out to measure whether practices and CCGs viewing OpenPrescribing already differ from their peers in prescribing behavior prior to using the tool.

**Prescribing Data Available on OpenPrescribing.**

1. A range of *prespecified prescribing measures* (eg, “proportion of statins that are high cost” and “antibiotic prescriptions per standardized population unit”).
2. A *price-per-unit* tool that uses a novel method developed at OpenPrescribing to identify a range of cost-saving opportunities bespoke for each institution, driven by analysis of national price variation within the same chemical and dose across each month. In essence, we calculate the mean price per unit (comparable to price-per-tablet) for every dose of every drug prescribed, for every practice and Clinical Commissioning Group (CCG); calculate the range of prices nationally; calculate the price per unit at the 10th centile practice or CCG; calculate the available savings for every organization, for every dose of every drug, were they to attain the price per unit of the 10th centile organization; rank these by total savings; and, then, present the highest value prescribing changes for each practice and CCG through a Web interface. Savings are, therefore, identified by a novel method using computationally intensive data analysis across the entire national prescribing dataset. At present, crucially for the analysis presented here, these savings can only be identified using OpenPrescribing [1]. Savings are best realized by viewing practice-level data.
3. A range of tools that collectively calculate the total money spent on items identified by NHS England as a *low priority* on the grounds of extremely low cost-effectiveness [2].

## Methods

### Prespecification and Protocol Registration

As this is an observational study of the impact of our own service, we endeavored to minimize any potential for conflict of interest impacting on results by fully prespecifying our methods and posting the protocol [8] and analytic code [9] on the Open Science Framework prior to commencement. In the protocol, we specified the outcomes to be measured along with the full analytic approach. The entire analytic code was written against a small sample of 7 institutions’ data, and we published it prior to conducting the analysis. There were no substantial changes to the outcomes or methodology (including the analytic code) between prespecification and reporting the results here.

### Data and Sources

We obtained the prescribing outcome data from the monthly prescribing dataset published by National Health Service (NHS) digital and aggregated by the OpenPrescribing project. The monthly prescribing datasets contain one row for each treatment and dose for each prescribing organization in NHS primary care in England, describing the number of prescriptions issued and the total cost. Each practice in England belongs to one of 207 CCGs; we aggregated practice data to these CCGs for CCG-level analyses. Practices with a very small list size (<1000) were excluded due to the likelihood of being an atypical practice.

We obtained data on the exposure (CCG and practice page views) from Google Analytics for OpenPrescribing.net. We collected page view data from the launch of the project (December 1, 2015) to the most recent data available at the time of extraction (January 14, 2018). Page view data contain the date of each view, along with information on which practice or CCG’s data were viewed and on whether any specific site features were viewed (eg, the price per unit or low-priority features). We aggregated page view dates to month-year.

### Exposures

The exposures used for this study are page views on the OpenPrescribing.net site. This is a proxy exposure, as we are not able to attribute site use to an individual practice or CCG, but instead assume that a high proportion of traffic to each practice’s or CCG’s prescribing page is from persons associated with that practice or CCG.

We generated two different exposure variables to determine the associations with viewing OpenPrescribing. First, for each month, we categorized each practice and CCG into one of the three categories: “not viewed”, during “month first viewed”, or “after first view”, according to when each practice or CCG was viewed on OpenPrescribing in relation that month. We used “not viewed” to classify the months before viewing a CCG or practice. We defined the “month first viewed” as the first month that a practice or CCG page was viewed more than once (in order to exclude one-off visits, which are unlikely to represent full engagement with the site) and “after first view” as every month after the “month first viewed,” until the end of follow-up. Second, we generated a variable to describe the total number of views of each practice and CCG page on OpenPrescribing (divided into categories: 0 views and tertiles of the number of practice views among those that were viewed). For the price-per-unit and low-priority outcomes (described below), the exposure was restricted to views of the price-per-unit and low-priority pages for an institution.

### Outcomes

We used 3 outcome variables in order to measure the effectiveness of the three main features of OpenPrescribing (see Textbox 1). First, we calculated mean percentile for each of the standard prespecified OpenPrescribing measures [10], excluding the NHS England low-priority measures (which are analyzed separately below) and those where a value judgment is not made (currently direct-acting oral anticoagulants [11] and two pregabalin measures [12,13]). We aggregated OpenPrescribing measure performance by taking the mean percentile across all included measures for each practice or CCG in each month. Second, we calculated price-per-unit efficiency as the total identified price-per-unit savings available for each practice and CCG in each month; full methods for calculating price-per-unit savings are described elsewhere [1]. We converted this into a rate per thousand patients per month using practice population denominators. Price-per-unit efficiency is a measure of potential savings. The measured outcome in this study is the difference in price-per-unit efficiency between time periods or exposure levels. Thirdly, we calculated total spending on NHS England low-priority measures (as described elsewhere [2]) for each practice and CCG in each month. We also converted this into a rate per thousand patients per month.

We measured outcomes on a monthly basis, over a period from 3 months before the launch of the respective tool (to obtain a suitable baseline) to the most recent available data. The launch dates for the outcomes were December 2015 for the OpenPrescribing measures (ie, the OpenPrescribing service as a whole), August 2017 for the price-per-unit feature, and September 2017 for the low-priority spend feature.

### Analysis

We described our analysis in detail in our prespecified analytic code, which has been shared in full [9]. Analyses were performed separately at practice and CCG levels. We used fixed-effects and between-effects panel regression in order to limit the measurement of variation within practice or CCG (ie, variation over time) and between practice and CCG, respectively.

#### Clinical Commissioning Group and Practice Views

In addition to the analysis prespecified in the protocol [9], we calculated summary statistics for the viewed number of practices and CCGs for each outcome in order to provide further context to the analysis.

#### Before and After Viewing

To measure the change in prescribing outcomes over time (within CCG or practice effects), we used a fixed-effects linear panel regression to remove the effect of time-invariant (between-practice) characteristics. We first used a univariable model and then added calendar month to the model in order to adjust for underlying national changes over time. This should leave only differences over time between practices that have and have not been viewed on OpenPrescribing.

#### Before Viewing

To measure differences between practices that have and have not been viewed on OpenPrescribing, we used between-effects linear panel regression. This was a simple univariable model to test the hypothesis, with the between-effects model removing any effects occurring over time. In order to remove any potential influence of OpenPrescribing, we used the 3-month period prior to the above launch dates for each outcome (as described in the outcomes section).

## Results

### Clinical Commissioning Group and Practice Views

Of the 207 CCGs included in the study, all were counted as exposed (≥2 views in the same month) for the mean measure outcome during at least 1 month, while 127 (61.4%) were viewed for the price-per-unit outcome and 68 (32.9%) for the low-priority prescribing outcome. We included 7318 practices in the study; of them, 4578 (62.56%) were viewed in at least 1 month for the mean measure outcome, 279 (3.81%) for the price-per-unit outcome, and 59 (0.81%) for the low-priority outcome.

### Prescribing Before and After Viewing OpenPrescribing

Table 1 shows the change in prescribing outcomes measured at CCG level during and after each CCG was first viewed on OpenPrescribing. Table 2 shows the same but at practice level. Univariable results include secular trends that exist regardless of any influence of OpenPrescribing. These crude unadjusted data are presented only for reference; multivariable results account for secular trends and show the impact of OpenPrescribing views. There was no change in mean OpenPrescribing measure score at either CCG or practice level. Although there was a significant change at practice level in the univariable analysis, this effect was eliminated by adjusting for the calendar month.

**Table 1:** Clinical Commissioning Group (CCG)-level results of fixed-effects linear panel regression showing the change in each outcome before and after the corresponding CCG page on OpenPrescribing.net was viewed. For the measure scores, higher is worse.

Outcome/Time period		Mean (SD)	Univariable		Adjusted for calendar month		
			Change	95% CI		Change	95% CI
**Mean measure score (%)**							
	Not viewed	50.1 (8.1)	Reference	N/A^a^		Reference	N/A
	Month first viewed	50.0 (8.4)	0.00	−0.24 to 0.23		−0.09	−0.42 to 0.23
	After first view	49.9 (8.5)	−0.05	−0.14 to 0.04		−0.26	−0.60 to 0.08
**Price per unit (£ per 1000 patients)**							
	Not viewed	351.19 (165.06)	Reference	N/A		Reference	N/A
	Month first viewed	335.94 (154.03)	−78.28	−92.32 to −64.24		−10.30	−21.87 to 1.26
	After first view	283.72 (135.58)	−132.75	−143.45 to −122.06		−14.70	−25.56 to −3.84
**Low-priority measures (£ per 1000 patients)**							
	Not viewed	191.68 (59.48)	Reference	N/A		Reference	N/A
	Month first viewed	192.79 (62.17)	−11.13	−15.33 to −6.93		−2.13	−6.41 to 2.15
	After first view	194.51 (64.40)	−9.94	−14.59 to −5.29		1.08	−3.91 to 6.07

^a^N/A: not applicable.

**Table 2:** Practice-level results of fixed-effects linear panel regression showing the change in each outcome before and after the corresponding practice page on OpenPrescribing.net was viewed. For the measure scores, higher is worse.

Outcome/Time period			Mean (SD)		Univariable			Adjusted for calendar month		
					Change		95% CI	Change		95% CI
**Mean measure score (%)**										
	Not viewed	45.8 (8.6)		Reference		N/A^a^		Reference	N/A	
	Month first viewed	46.0 (8.8)		−0.35		−0.46 to −0.24		−0.07	−0.18 to 0.05	
	After first view	46.0 (8.8)		−0.52		−0.57 to −0.47		−0.04	−0.10 to 0.02	
**Price per unit (£ per 1000 patients)**										
	Not viewed	426.86 (251.99)		Reference		N/A		Reference	N/A	
	Month first viewed	419.51 (229.25)		−98.57		−116.96 to −80.18		−31.49	−48.12 to −14.86	
	After first view	385.70 (220.13)		−148.36		−163.26 to −133.46		−40.42	−54.04 to −26.81	
**Low-priority measures (£ per 1000 patients)**										
	Not viewed	185.35 (154.91)		Reference		N/A		Reference	N/A	
	Month first viewed	234.62 (205.06)		−12.84		−34.87 to 9.18		−4.33	−26.32 to 17.66	
	After first view	250.62 (223.00)		−13.15		−41.05 to 14.74		−3.11	−30.97 to 24.76	

^a^N/A: not applicable.

There was a reduction in available price-per-unit savings after CCGs (Table 1) and practices (Table 2) were viewed on OpenPrescribing. The effect size was greater at practice level (−£40.42 per thousand patients per month; 95% CI −54.04 to −26.81) than at CCG level (−£14.70 per thousand patients per month; 95% CI −25.56 to −3.84). In the univariable analysis, there was a much greater effect size due to the overall trend of decreasing available savings over the study period, but the effect remained after adjustment for the calendar month.

Multiplying the estimated (per thousand patient) saving by the CCG and practice populations in the “after looking” months gives a total estimated saving of £1.47 million (95% CI £384 thousand to £2.56 million) at CCG level and £243 thousand (95% CI £162 thousand to £326 thousand) at practice level in practices and CCGs whose data were viewed. It is possible that some of these savings will overlap, and therefore, it is not appropriate to add the two figures together to create total savings. Extrapolating these savings figures to all CCGs and practices across England, if all institutions’ data were viewed, it would generate an estimated annual saving of £9.7 million at CCG level and £26.8 million at practice level.

**Table 3:** Clinical Commissioning Group (CCG)-level results of between-effects linear panel regression, showing the differences in each outcome, before each feature was launched, between CCG pages that were subsequently viewed on OpenPrescribing.net at various levels and those that were not. For the measure scores, higher is worse.

Outcome/Number of views			CCGs, n (%)		Mean (SD)		Change		95% CI
**Mean measure score (%)**									
	0	0 (0)		N/A^a^		N/A		N/A	
	32-135	69 (33.5)		50.6 (8.2)		Reference		N/A	
	139-228	70 (34.0)		50.4 (7.5)		−0.20		−2.93 to .52	
	231-2219	67 (32.5)		48.9 (8.7)		−1.74		−4.50 to 1.01	
**Price per** **unit** **(£ per 1000 patients)**									
	0	40 (19.3)		365.67 (130.69)		Reference		N/A	
	1-3	66 (31.9)		366.31 (154.46)		0.64		−61.16 to 62.43	
	4-9	47 (22.7)		416.62 (186.29)		50.95		−15.40 to 117.29	
	10-137	54 (26.1)		444.80 (169.25)		79.12		14.79 to 143.46	
**Low-priority measures** **(£ per 1000 patients)**									
	0	55 (26.6)		198.19 (52.31)		Reference		N/A	
	1	57 (27.5)		200.15 (59.52)		1.96		−20.40 to 24.32	
	2-4	47 (22.7)		193.84 (55.02)		−4.36		−27.85 to 19.14	
	5-52	48 (23.2)		196.76 (75.82)		−1.44		−24.80 to 21.93	

^a^N/A: not applicable.

**Table 4:** Practice-level results of between-effects linear panel regression, showing the differences in each outcome, before each feature was launched, between practice pages that were subsequently viewed on OpenPrescribing.net at various levels and those that were not. For the measure scores, higher is worse.

Outcome/Number of views		Practices, n (%)	Mean (SD)	Change	95% CI
**Mean measure score** **(%)**					
	0	381 (5.0)	45.6 (8.5)	Reference	N/A^a^
	1-4	3024 (39.3)	45.7 (8.5)	0.11	−0.76 to 0.99
	5-8	2013 (26.2)	46.1 (8.4)	0.49	−0.41 to 1.39
	9-343	2278 (29.6)	46.7 (8.3)	1.21	0.32 to 2.10
**Price per** **unit** **(£ per 1000 patients)**					
	0	6695 (91.6)	477.29 (259.28)	Reference	N/A
	1	298 (4.1)	480.02 (262.51)	3.95	−24.62 to 32.52
	2	114 (1.6)	544.99 (315.40)	69.42	23.84 to 114.99
	3-49	205 (2.8)	524.52 (383.27)	47.59	13.38 to 81.81
**Low-priority measures** **(£ per 1000 patients)**					
	0	6891 (94.2)	187.72 (155.65)	Reference	N/A
	1	308 (4.2)	229.80 (175.17)	41.88	25.42 to 58.35
	2-26	119 (1.6)	251.37 (240.24)	63.94	37.81 to 90.08
	Insufficient views for additional tertile	N/A	N/A	N/A	N/A

^a^N/A: not applicable.

The total “available” savings calculated by the tool for the time after CCGs or practices were viewed were £31.3 million at CCG level and £2.4 million at practice level. In our paper [1], we estimated that around half of these “available” savings might be “achievable.” This means that around 10% of the achievable savings were realized at CCG level and around 20% realized at practice level.There was no change in the total spend on low-priority measures at CCG or practice level. The small reduction seen at CCG level in the univariable analysis is again eliminated after adjustment for the calendar month.

### Pre-Existing Differences Between Practices That Have or Have Not Been Viewed

Table 3 shows the differences in prescribing outcomes before each service was launched, at CCG level, stratified according to the level of views after the service was launched for each CCG. Table 4 shows the same at practice level. CCGs that have been viewed had higher available price-per-unit savings (ie, they were less cost-efficient as prescribers) but were similar for the other two outcome measures. For practices, those that have been viewed were worse for price per unit and low-priority spending but similar with respect to the mean measure score.

## Discussion

### Principal Findings

We found that the total available price-per-unit savings decreased following views of OpenPrescribing. This saving corresponds to a total measured decrease in spend of £243 thousand at practice level and £1.47 million at CCG level, between the feature launch and end of follow-up (August to November 2017), for practices and CCGs where the tool was viewed. We found a greater saving per patient for the practice-level exposure, but a higher overall estimated saving at CCG level, due to a greater exposed population at the CCG level. Our analytic methods make every effort to remove confounding effects, such as differences between institutions or national secular trends. Extrapolating the observed savings nationally would generate a total saving of £9.7 million per year at CCG level and £26.8 million per year at practice level. Savings from this new tool were calculated with only 1 to 3 months of follow-up data and may increase over time. We did not find any changes in the overall prescribing score or low-priority measure spend; possible reasons are discussed below. Contrary to expectations, we found that institutions whose data were viewed on OpenPrescribing were overall performing more poorly on prescribing measures prior to viewing.

### Strengths and Weaknesses

We were able to remove between-practice (time-invariant) confounding effects with the use of fixed-effects linear panel regression. This meant that only differences occurring over time, before and after viewing, were measured. In addition to this, adjusting for calendar month allowed us to appropriately remove the effect of any overall national trends over time, which are independent of any effect that OpenPrescribing might have. There is a theoretical possibility of there being a systematic difference between the underlying secular trends between exposed and unexposed practices. However, for the outcome where we saw an effect (price per unit), practitioners can only identify savings by using our tool, as this is a novel method of identifying savings, requiring computationally intensive calculations setting the individual CCG or practice’s prescribing in the context of all other organizations’ prescribing for every drug-dose pair. This, therefore, very strongly militates against any such possibility of confounding.

By fully prespecifying our methods and publishing our protocol and analytic code in advance of conducting our analysis, we reduced, as far as possible, the impact of any potential conflict of interest. We were only able to use a proxy indicator of each institution’s use of the OpenPrescribing tools, since we cannot determine who exactly is viewing the website, only that a given institution’s pages have been viewed. We can identify some traffic that originates from NHS IP (Interet Protocol) addresses, and anecdotally, we receive regular feedback and queries from NHS users. However, we are not able to reliably estimate the proportion of traffic that is from NHS use. This is because not all NHS organizations use an NHS internet protocol address, and some traffic is likely to be from NHS users accessing the site from private or mobile internet connections. This is likely to have added noise to the data and, as a result, is likely to have led to our underestimating any impact from the tools, as persons not associated with a practice or CCG can view the site (meaning an institution is incorrectly counted as exposed) but cannot impact on prescribing choices. It is difficult to estimate how great this effect might be.

The number of views for each feature on the site varied substantially, largely because newer tools (such as price per unit and low-priority measures) have only been available for a short period. This means it was only possible to measure the effects on a relatively small proportion of all practices for these tools. In addition, we specified the start time for possible impact on the standard prespecified prescribing measures as December 2015 because this is when the OpenPrescribing site launched. However, very few measures were available at the initial site launch, and these were added gradually over the following 2 years. This substantially reduced our ability to detect an impact on the standard prescribing measures; however, there are no methodologically straightforward means to account for this variation in the characteristics of the exposure over time.

### Findings in Context

There are many providers of services related to medicines optimization [3-6]. Such providers make varying degrees of claims, with some just being a description of the services provided, while others make strong claims of efficacy. For example, ScriptSwitch (a point-of-care tool that makes automated suggestions of preferred alternative medication options when a prescription is initiated) claims to have “delivered over £50 million in savings to the NHS” [4], but it is not obvious how this was measured or over what period these savings were made. Another example is from Oracle (a commercial database vendor whose software is used by NHS Business Services Authority to make prescribing data available to a small number of NHS users with passwords), which claims that it has “enabled antibiotic prescribing to be reduced by 7%” [14,15]. However, we are aware of no evidence being given for this very substantial claim, which must be interpreted in the context of a pre-existing downward trend in antibiotic prescribing [16], and extensive work to reduce antibiotic prescribing following the Chief Medical Officer’s prioritization of the issue in 2013 [17,18]. Lastly, Prescribing Services (“Risk Stratification in Prescribing & Screening” tool) have published an attempt to measure the impact of its tool and claimed a reduction in emergency admissions; however, the methodology used is not described in any detail [19].

### Policy Implications and Interpretation

We found a substantial positive impact from the use of our prescribing data tool. We also show that it is possible to conduct a robust analysis of the impact of such a tool. We will continue to monitor the effectiveness of existing and new features in order to refine the tools and monitor impact. In our view, commissioners of health services should expect robust evaluations, conducted pragmatically and at low cost at the point of care, for all such digital tools making claims for positive impact on population health and cost-effectiveness.

We found the price-per-unit savings to be higher per patient at practice level than CCG level; this is to be expected, as tailored prescribing changes from this tool are best identified at the level of individual practices, as discussed in prior work [1]. However, these savings might be achieved more simply and efficiently through a national policy change.

The lack of a positive effect on the overall prescribing measure might be explained, in part, by the construction of the mean score. There are 34 different measures making up the mean score, so improvements made by a practice or CCG focusing on one measure, in particular, is likely to be drowned out by the noise of other measures. While we would like to have measured the impact on each measure individually, this would make the analysis extremely complex due to the variety between measures and would also make interpretation more difficult. It would have been preferable to use a more parametric method to summarize the measures, such as mean Z-score, but this was not possible due to nonnormal distributions (eg, bimodal). Additionally, in contrast with the price-per-unit outcome, where OpenPrescribing is the only known source of determining such savings, it is possible that many practices and CCGs are already aware of many of the issues and had existing work to improve prescribing independently of OpenPrescribing use. This is true for many of the standard OpenPrescribing measures and for the NHS England low-priority measures, which obtained some publicity when they were announced. Other possible reasons for the lack of effect with the low-priority outcome are the potential for changes in one type of prescribing to be lost in the noise of the others and the lack of follow-up time since launch (at most 3 months).

Prior to this analysis, we hypothesized that practices or CCGs that looked at OpenPrescribing might already have better overall prescribing than those who do not, on the grounds that institutions who are proactively engaged with their data are likely also to be more effective at improving their prescribing. In fact, we found that the opposite is true for some outcomes. However, this may reflect the way the OpenPrescribing site operates, in that various features highlight practices that are performing the least well on specific prescribing measures; for example, when examining the performance on one measure for all practices in a CCG, practices are ordered from worst to best, increasing the likelihood that worse practices will be clicked on when browsing. Similarly, the OpenPrescribing “email alerts” service uses various statistical process control methods to highlight specific practices and CCGs with worse prescribing [20].

### Conclusions

We found a clinically significant positive impact from the use of our prescribing data tool. We also show that it is possible to conduct a robust analysis of the impact of such a tool. We will continue to monitor the performance of the OpenPrescribing services as more follow-up time accrues and as features are added and enhanced. Our methods and full prespecification may represent a good template for similar impact assessments on services that aim to improve health care.

## Abbreviations

CCG: Clinical Commissioning Group
NHS: National Health Service
